# Evaluation of Plasmatic Procalcitonin in Healthy, and in Systemic Inflammatory Response Syndrome (SIRS) Negative or Positive Colic Horses

**DOI:** 10.3390/ani11072015

**Published:** 2021-07-06

**Authors:** Irene Nocera, Francesca Bonelli, Valentina Vitale, Valentina Meucci, Giuseppe Conte, Eduard Jose-Cunilleras, Luis Alfonso Gracia-Calvo, Micaela Sgorbini

**Affiliations:** 1 Department of Veterinary Sciences, Veterinary Teaching Hospital “Mario Modenato”, via Livornese snc, San Piero a Grado, 56124 Pisa, Italy; francesca.bonelli@unipi.it (F.B.); v.vitale_vet@yahoo.es (V.V.); valentina.meucci@unipi.it (V.M.); micaela.sgorbini@unipi.it (M.S.); 2 Department of Agriculture, Food, Environment, University of Pisa, via del Borghetto, 80, 56124 Pisa, Italy; giuseppe.conte@unipi.it; 3 Equine Internal Medicine Service, Veterinary Teaching Hospital, Department of Animal Medicine and Surgery, Faculty of Veterinary Medicine, Autonomous University of Barcelona, 08193 Barcelona, Spain; eduard.jose.cunilleras@uab.cat; 4 Veterinary Teaching Hospital, University of Helsinki, Koetilankuja 1, 00790 Helsinki, Finland; luis.a.graciacalvo@helsinki.fi

**Keywords:** horse, colic, procalcitonin, systemic inflammatory response syndrome

## Abstract

**Simple Summary:**

Procalcitonin (PCT) increased in the case of systemic inflammatory response syndrome (SIRS), especially due to bacterial infection. The correlation between SIRS score and plasma PCT levels in horses have not been evaluated, and no studies investigated plasma PCT concentration over time. In the present study, PCT and SIRS score were evaluated in colic horses at admission to the hospital and at 24, 48, 72 and 96 h. Statistically differences were detected between healthy vs. all colic horses and between healthy vs. SIRS positive or SIRS negative horses. No correlation was observed between SIRS score and PCT. This suggests a role of plasmatic PCT as good biomarker for colic.

**Abstract:**

Colic horses show systemic inflammatory response syndrome (SIRS) clinical signs. Procalcitonin (PCT) showed increased circulating levels in sick horses. This study compares plasma PCT concentrations in healthy vs. SIRS negative/positive colic horses over time, and evaluates PCT and SIRS score potential correlation, to verify the usefulness of PCT for the evaluation of SIRS severity. Ninety-one horses were included; 43/91 were healthy, on basis of physical examination, blood work and SIRS score (score = 0), while 48/91 were sick colic horses, classified as SIRS-negative (score < 2) and positive (score ≥ 2). Moreover, a 0–6 point-scale SIRS score was calculated (assessing mucous membrane color and blood lactate concentration). PCT was evaluated at admission, and at 24, 48, 72 and 96 h, using a commercial kit for equine species. We verified by the ANOVA test PCT differences between healthy vs. colic horses, healthy vs. SIRS-negative or SIRS-positive colic horses, at all sampling times, and the correlation between the SIRS score at admission with the SIRS score. Statistically significant differences were detected between healthy vs. all colic horses and between healthy vs. SIRS-positive or negative horses at all sampling times. No correlation was observed between the SIRS score at admission and PCT values. PCT was statistically higher in colic horses compared to the healthy ones, suggesting a role as a biomarker for colic.

## 1. Introduction

Colic is a term used to describe abdominal pain and represents the most common reason for emergency veterinary treatment. Acute gastrointestinal disease is the most common cause of colic in horses, but a wide range of different diseases affecting the abdominal organs, such as urinary or genital tract disorders, can also be reasons for horses showing signs of colic [1]. Many horses with colic show clinical signs similar to those described for systemic inflammatory response syndrome (SIRS) [2]. Previous studies indicate that the clinical pattern associated with inflammatory intestinal diseases or strangulating obstructions are more accurately described by the term SIRS instead of endotoxemia [2,3]. Recently, Roy and colleagues [4] evaluated a model of severe SIRS to predict outcome, which included the historic SIRS criteria already used, plus the evaluation of the blood lactate concentration and mucous membrane status. The study showed that horses with three and four signs of SIRS had increased odds of death compared to non-SIRS cases or the horses with just two signs of SIRS. 

The biggest challenge of an equine patient affected by SIRS is early treatment. Thus, an early diagnosis of this condition may lead to a prompt and effective therapy and to better prognosis [5,6]. The literature reported that the criteria used to diagnose SIRS in adult horses were fever, tachycardia, tachypnoea, and an abnormal white blood cell count [7]. In a recent review, the criteria used for the diagnosis of SIRS did not provide information regarding the etiology of the disease because of their lack of specificity [3]. Moreover, the review showed that these criteria are too sensitive, leading to the misclassification of sick patients in clinical settings. Biomarkers played a role as efficient diagnostic tools in clinical settings, and they have been investigated in veterinary medicine, owing to the great expansion of available technology [8]. The National Institute of Health Biomarkers Definitions Working Group defined an ideal biomarker as being “a more rapid diagnostic tools, to be able to discriminate between normal or pathologic process, and to give information about response to a pharmacologic or otherwise therapeutic intervention” [9]. Concerning SIRS, diagnostic and prognostic biomarkers have the potential to replace the overly sensitive yet poorly specific SIRS score criteria and represent a promising and fertile research field for veterinary diseases, including equine colic. Due to the promising results obtained in human medicine, Procalcitonin (PCT) has been recently investigated in veterinary species. Serum PCT concentrations in healthy patients are very low due to the restriction of the CALC-I gene transcription by the neuroendocrine cells in the thyroid gland and in the lungs [6,10]. In the case of inflammation due to bacteria, the expression of the CALC-I gene is up-regulated in many cell types, leading to the release of PCT into the circulation and to the increase in its serum levels [6,10,11,12]. Adult horses, foals and cattle showed increased circulating levels of PCT during pathological conditions caused by bacteria or by the translocation of bacteria and/or their products into the bloodstream [13,14,15,16,17,18,19,20,21]. A Dutch study showed that naturally endotoxemic horses affected by colic had higher PCT plasma levels compared to the healthy control group [15]. Two more papers evaluated PCT in horses affected by colic: in 2015, Bonelli and colleagues evaluated plasma PCT concentrations in a population of healthy (*n* = 30) and SIRS horses (*n* = 48), showing that PCT levels were increased in sick horses [14]. A recent study evaluated PCT concentrations in both the plasma and peritoneal fluid of colic horses, and found a higher peritoneal PCT concentration in sick animals compared to the healthy ones [21]. To the best of our knowledge, no studies evaluated the correlation between the SIRS score and plasma PCT levels, and no studies investigated plasma PCT concentration at different sampling times of hospitalization in colic horses. 

Our hypothesis is that PCT could be a good biomarker for assessing the severity of equine colic and for recognizing the SIRS status of patients admitted to veterinary clinics. Thus, the aims of this study were to evaluate the potential differences in plasma PCT concentrations between healthy vs. SIRS-negative or SIRS-positive horses in order to understand if PCT could be a useful biomarker to distinguish between these conditions and drive clinical decisions of equine veterinarians. Moreover, PCT concentrations throughout the hospitalized period were evaluated. The second aim was to evaluate the potential relationship between PCT and SIRS score in order to verify if this biomarker could be useful for the evaluation of SIRS severity.

## 2. Materials and Methods

The present in vivo prospective multicentric study was approved by the Institutional Animal Care and Use Committee of the University of Pisa (Prot. N. 2473/14) and an owner’s written consent was obtained for the collection of plasma for all the horses included in this study. The study was performed over a three-year period.

### 2.1. Study Population

A total of 91 adult horses were included in the present study. Forty-three out of ninety-one (47.3%) were healthy adult horses enrolled in reproduction activity, while 48/91 (52.7%) were sick adult colic horses, referred to three different veterinary teaching hospitals (VTHs) providing secondary health care. 

At admission time, all the horses underwent a complete physical examination and blood work analysis, plus additional ancillary diagnostic procedures if needed. Moreover, the following data were recorded in order to evaluate the SIRS positivity/negativity and score [3]: the presence of abnormal leukocyte count or distribution as leukopenia, leukocytosis or >10% band neutrophils (lower than 5 or higher than 12.5 × 10^3^/μL), hyperthermia or hypothermia (lower than 37 or higher than 38.5 °C), tachycardia (>52 bpm), tachypnea (>20 bpm). The SIRS score evaluation was made by expert veterinarians previously trained for this assessment (I.N. and V.V.). 

Those animals with a normal physical examination, laboratory data within reference ranges and a SIRS-negative score (score = 0) were included in the control group. According to the literature [3], sick colic horses with 0 or 1 abnormal criterion were considered SIRS-negative, while sick colic horses with 2 or more abnormal criteria were included in the SIRS-positive group. Moreover, the blood lactate concentration (normal blood lactate: ≤ 2.06 mmol/L vs. abnormal > 2.06 mmol/L) and color of the mucous membranes (normal mucous membranes: pink and moist vs. abnormal mucous membranes: injected, purple, muddy, toxic, red, or white) were evaluated to calculate the 0–6 point-scale SIRS score [4].

Along with clinical data, blood samples were collected for Complete Blood Count (CBC) and PCT analysis from the jugular vein of each animal included, using a sterile syringe and 16G needle. Samples were drawn once in healthy horses, while at admission time (T0), then 24 (T1), 48 (T2), 72 (T3), 96 (T4) hours after admission to the VTHs in colic horses. After the first sampling (T0), all the horses received appropriate treatments based on the clinical signs and the diagnosis. All blood samples were divided in two aliquots: 1 mL in aliquot was collected in a potassium ethylene diamine tetra acetic (K2EDTA) test tube and analyzed by a cell counter (ProCyte Dx™, IDEXX Laboratories Italia s.r.l., Milan, Italy) within 5 min after the collection. A second 2.5 mL aliquot was collected in LH-heparin tubes and centrifuged at 3000 rpm for 10 min within 30 min of collection. The harvested plasma was placed in sterile tubes, and frozen at −20 °C. PCT was measured in a single batch, as described below. 

The time elapsed from the onset of clinical signs and the time of admission and first sampling or to collect a complete history regarding the pre-admission treatments were not standardized. Moreover, some colic horses were discharged or euthanized before T4, so it was not possible to collect samples through the whole study period (T0–T4) for all the patients included.

### 2.2. PCT Evaluation

PCT concentrations were determined as previously reported [13,14] with a commercial kit for equine species (Horse Procalcitonin ELISA kit, MyBiosource.com, Inc., San Diego, CA, USA). 

The intra-assay coefficient of variation was determined from 10 replicates of equine plasma samples containing low and high PCT concentrations. These samples were obtained by the addition of standard PCT in equine blank samples. The inter-assay coefficient of variation was determined from values obtained by repeating the analysis of duplicate samples with low and high PCT concentrations in 5 different assays. To establish the detection limit for equine plasma PCT, we performed repeated PCT measurements using equine samples with low PCT concentrations (<10.0 pg/mL). Samples were measured in 10 replicates in a single assay and in 5 different assays. The intra- and inter-assay coefficient of variations were both < 15%, and the limit of detection of the method was 10 pg/mL.

### 2.3. Statistical Analysis

Data were analyzed using a commercial software (JMP, SAS Institute Inc., Cary, NC, USA). Due to the small number of samples, the PCT concentration data were transformed into logarithmic values in order to obtain a normal distribution, as demonstrated by the Shapiro–Wilk test (*p* > 0.05). The results were then expressed as the Least Square Mean (LSM) mean and mean standard error (MSE). The transformed values were analyzed using an Analysis of Variance (ANOVA) with the following linear model:

y_ijk_ = μ + G_i_ + T_j_ + e_ijk_

where: y_ijk_ = Log PCT; μ = mean; G_i_ = fixed effect of i^th^ group (healthy vs. total colic horses; healthy vs. SIRS-negative; healthy vs. SIRS-positive horses); T_j_ = the fixed effect of the j^th^ time period (T0, T1, T2, T3, T4); e_ijk_ = residual error. Statistical difference was set at *p* < 0.05. In cases of a significant effect, a post hoc HSD analysis of Tukey was performed.

The Pearson correlation coefficient was calculated to assess the relationship between the SIRS score and the PCT concentration at T0.

## 3. Results

Six out of forty-three (14%) healthy horses were stallions, 9/43 (20.9%) geldings, and 28/43 (65.1%) females. The median age was 6 years old (mean 7.9 years old; range 2–20 years old; IQR 4–12.2 years old). The horses were of different breeds: Standardbred (*n* = 20), Warmblood (*n* = 15), Thoroughbred (*n* = 5), and Arabian (*n* = 3).

Nine out of forty-eight (18.7%) colic horses were stallions, 20/48 (41.7%) geldings, and 19/48 (39.6%) females. Median age was 11.5 years old (mean 11.8 years old; range 3 mo-32 years old; IQR 6–19 years old). The horses were of different breeds: cross-breed (*n* = 10), Warmblood (*n* = 9), Ponies (*n* = 6), Pure Spanish Horse (*n* = 5), Quarter Horse (*n* = 4), Arabian (*n* = 4), Thoroughbred (*n* = 3), Standardbred (*n* = 3), and Draft horses (*n* = 4).

The results concerning the diagnosis (grouped as non-strangulating or strangulating colic) and treatment of the total of colic horses, the SIRS-negative colic horses and the SIRS-positive colic horses are reported in Table 1.

Regarding the SIRS score, 5/48 (10.4%) horses had SIRS score = 0, 12/48 (25%) SIRS score = 1, 9/48 (18.8%) SIRS score = 2, 7/48 (14.6%) had a SIRS score = 3, 10/48 (20.8%) had SIRS score = 4, 4/48 (8.3%) a SIRS score = 5, and 1/48 (2.1%) horse had a SIRS score = 6. 

None of the horses spontaneously died, while 44/48 (91.7%) horses were discharged and 4/48 (8.3%) were humanely euthanized for poor prognosis. The diagnosis (grouped as non-strangulating or strangulating colic) of the discharged or euthanized horses and the SIRS status (positive or negative) are reported in Table 2.

Seven out of forty-four (15.9%) (all obstructive non-strangulating colic) horses were discharged after the first sampling, 4/44 (9.1%) (3/4 obstructive non-strangulating and 1/4 strangulating colic) after the second and the third, respectively, 8/44 (18.2%) (all obstructive non-strangulating colic) after the fourth and 21/44 (47.7%) after the last sampling (11/21 obstructive non-strangulating and 10/21 obstructive strangulating colic). Two out of four (50%) horses were euthanized after the first sampling (both obstructive strangulating colic) and 2/4 horses (50%) were euthanized after the last sampling (1/4 obstructive non-strangulating and 1/4 obstructive strangulating colic). 

The logarithmic value of plasma PCT concentration obtained in healthy, total colic horses, SIRS-negative, and SIRS-positive horses are reported in Table S1. 

**Figure 1:**
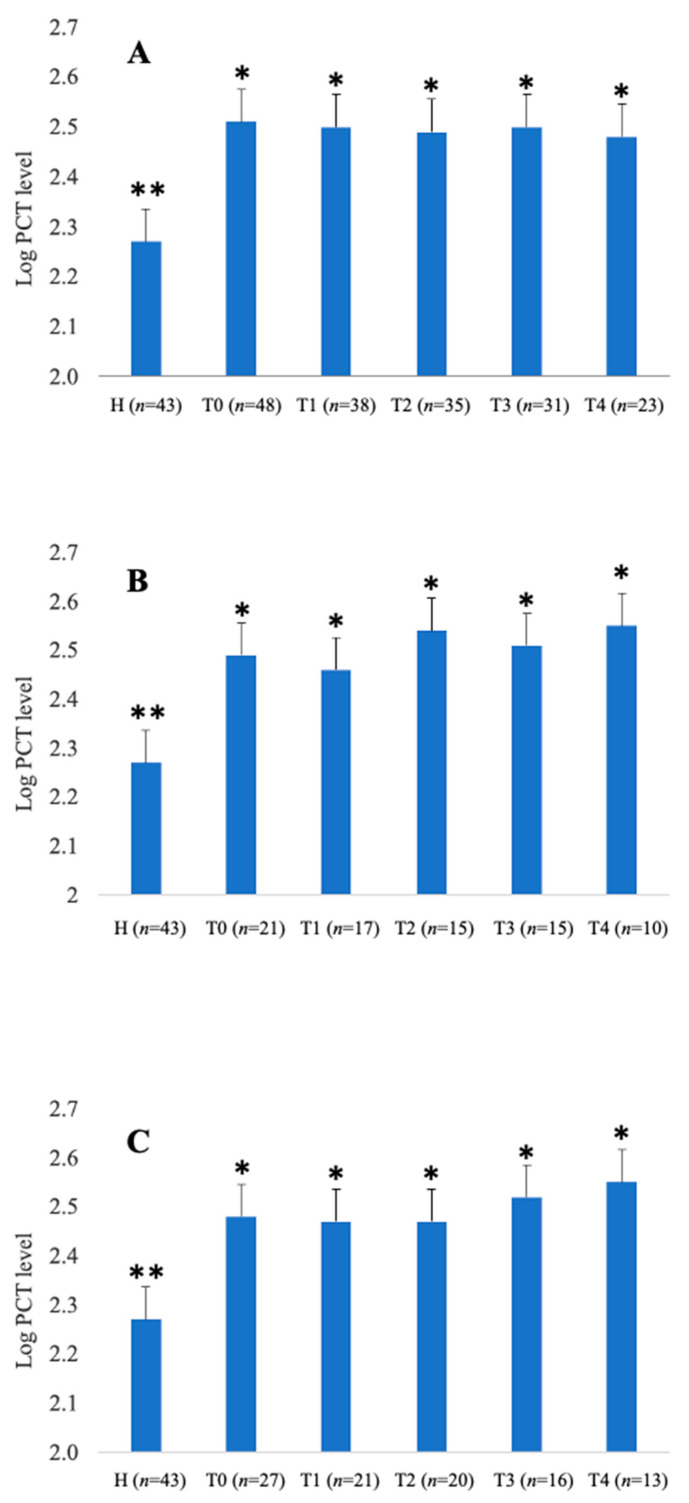
Logarithmic values of plasma PCT concentrations in healthy (**H**) and total colic horses (**A**), SIRS-positive colic horses (**B**), and SIRS-negative horses (**C**) at admission time (T0), then 24 (T1), 48 (T2), 72 (T3) and 96 (T4) hours after admission. The different upper-case symbols above the bar charts denote a significant difference from healthy group (** ≠ *: *p* < 0.05).

Statistically significant differences were detected between healthy vs. all colic horses (*p* = 0.048) (Figure 1A) and between healthy vs. SIRS-positive (Figure 1B) or SIRS-negative (Figure 1C) horses (*p* = 0.048) at all sampling times (T0–T96). No correlation was observed between the SIRS score and plasma PCT values (r = 0.061, *p* = 0.63).

## 4. Discussion

Procalcitonin (PCT) has been studied over the last few years as a biomarker of inflammation and/or infection, both in human [22] and in large animal species [13,14,15,16,17,18,19,20,21,23,24,25,26]. The aims of this study were to evaluate the potential differences in plasma PCT concentrations between healthy vs. SIRS-negative vs. SIRS-positive horses in order to understand if PCT can be a useful biomarker to distinguish between these conditions and drive clinical decisions of equine veterinarians and SIRS score in order to verify if this biomarker could be useful for the evaluation of SIRS severity. Plasma PCT levels throughout colic hospitalization were also investigated.

In this study, we found differences between healthy vs. total colic horses at admission time. Our results are in line with previous studies that reported differences between sick vs. healthy bovine [16,18,20,25], horses [14,15,19,24,26] and foals [13] affected by different diseases. 

To date, there are two studies published on the evaluation of PCT concentrations in healthy and colic horses [15,21] reporting that the control and the sick population presented statistically significant differences. Our results are in line with the literature, showing that plasma PCT concentrations were statistically higher in colic horses compared to the healthy ones. Moreover, our study found that healthy horses presented statistically lower plasma PCT concentrations compared to both SIRS-positive and SIRS-negative colic horses. If we compare the PCT values obtained, we are in line with the more recent one performed on strangulating intestinal lesions [21] while our results are lower compared to Teschener and colleagues [15]. This difference may be related to the different laboratory method applied by the Dutch paper, in which an ELISA method developed in 2014 [27] was used.

Data concerning the difference in plasma PCT levels between healthy and SIRS-positive horses at the admission time (T0) are in line with the hypothesis that PCT could be considered a good biomarker for the assessment of equine hospitalization. During a colic episode, clinical signs of SIRS are mostly related to the release of lipopolysaccharide (LPS) from the gut to the blood stream, leading to a systemic inflammatory pattern [2]. Our results evaluating PCT levels over time showed that PCT remained higher in colic horses compared the healthy ones until 96 h after admission at the VTHs. The kinetic pattern of plasmatic PCT after LPS infusion has been studied in humans [28,29] and in horses [17]. The plasma PCT concentration peaked 60 min after LPS administration and remained stable for 24 h; unfortunately, no data showed the PCT kinetic pattern after LPS infusion for a longer period in horses. The literature in laboratory animals and humans reported that PCT levels tend to decrease as soon as the pathogen and the related inflammatory status is controlled by the immune system [10]. The 2016 Surviving Sepsis Campaign (SSC) guidelines proposed that the serial measurement of PCT concentrations can be used to support shortening the duration of antimicrobial therapy in sepsis patients [30]. To date, several meta-analyses have assessed the value of PCT to guide the therapy in intensive care unit patients [31]. Further studies evaluating plasma PCT levels in colic horses for a longer period would be needed in order to assess whether this biomarker could be used to guide the therapy as in humans.

We found no correlation between the SIRS score and plasma PCT concentrations. This result was surprising, but some explanations may be found. As previously mentioned, the SIRS score often lacks specificity and its criteria are too sensitive, leading to the misclassification of sick patients in clinical settings [3,7]. Despite the SIRS score is widely used in the equine literature for classifying horses as healthy or sick, the limitation of the SIRS score might have influenced our results. Moreover, the nature and the severity of the pathological condition should also be considered. All the VTHs involved in the study provide secondary health care, meaning that the severity of the colic cases at admission time not only depended on the etiology of the colic (i.e., surgical or medical colic, strangulating or non-strangulating colic), but also on the timing of referral to the clinic; a wide difference in treatments received in the field should also be considered as an effect which could have influenced PCT levels at admission time. Further studies would be needed in order to include a wider population; also, enrolling colic cases referred at the VTHs with no field treatment received, would make it clearer whether PCT could have a role in diagnosing different type of colic, or not. Despite the etiology of the clinical problem, horses with colic are usually referred at the VTHs for the severity of their conditions. This means that PCT would be considered a useful biomarker in the clinical practice even without the possibility of differentiating the different type of colic.

A limitation of the present study is the lack of SIRS score evaluation over time and the lack of information about specific treatments applied. Further studies will correlate plasma PCT concentrations upon admission time and throughout the hospitalization, aiming to find a possible relation with the biomarker and the response to treatment. This would be very interesting in the evaluation of whether PCT could play the same role as in human medicine in guiding the therapy at intensive care units. 

## 5. Conclusions

In conclusion, based on the results of the present study, PCT could be considered a good biomarker to distinguish horses referred for colic from healthy ones. Further studies would be needed in order to increase the population investigated and to find a cut-off value which could differentiate between the different pathological conditions that can cause colic syndrome in horses.

## Figures and Tables

**Table 1:** Results concerning the diagnosis (grouped as non-strangulating or strangulating colic) and treatment of the total of colic horses, the SIRS-negative colic horses and the SIRS-positive colic horses enrolled in the study.

Colic Diagnosis	Total Colic 48/91 (52.7%)	
obstructive non-strangulating colic	34/48 (70.8%)	22/34 treated medically 12/24 treated surgically
obstructive strangulating colic	14/48 (29.2%)	14/14 treated surgically
	SIRS-negative colic 27/48 (56%)	
obstructive non-strangulating colic	19/27 (70.4%)	13/19 medically 6/19 surgically
obstructive strangulating colic	8/27 (29.6%)	8/8 surgically
	SIRS-positive colic 21/48 (43.7%)	
obstructive non-strangulating colic	15/21 (71.4%)	9/15 medically 6/15 surgically
obstructive strangulating colic	6/21 (28.6%)	6/6 surgically

**Table 2:** Diagnosis (grouped as non-strangulating or strangulating colic) and SIRS status (positive or negative) of the discharged or euthanized horses.

Colic Diagnosis	Discharged 44/48 (91.7%)		Euthanized 4/48 (8.3%)	
	SIRS-Negative 26/44 (59.1%)	SIRS-Positive 18/44 (40.9%)	SIRS-Negative 1/4 (25%)	SIRS-Positive 3/4 (74%)
obstructive non-strangulating colic	33/44 (75%)		1/4 (25%)	
obstructive strangulating colic	11/44 (25%)		3/4 (75%)	

## Data Availability

The data presented in this study are available on request from the corresponding author.

## Supplementary Materials

The following are available online at https://www.mdpi.com/article/10.3390/ani11072015/s1, Table S1: Logarithmic value of plasma procalcitonin (PCT) concentrations (pg/mL) in healthy horses, total colic, systemic inflammatory response syndrome (SIRS) negative and SIRS positive colic horses at admission time (T0), then 24 (T1), 48 (T2), 72 (T3) and 96 (T4) hours after admission. Within row, different superscripts denote a significant difference (a ≠ b: *p* < 0.05).
