# Supplementary material for: Evaluation of Plasmatic Procalcitonin in Healthy, and in Systemic Inflammatory Response Syndrome (SIRS) Negative or Positive Colic Horses
Source: Animals (Basel). 2021 Jul 6;11(7):2015. doi: 10.3390/ani11072015 (PMC8300415; doi:10.3390/ani11072015)
Supplement: Supplementary file 1 [file animals-11-02015-s001.zip › animals-1251629-supplementary.pdf]

Table S1. Logarithmic value of plasma procalcitonin (PCT) concentrations (pg/mL) in healthy horses, total colic, systemic inflammatory response syndrome (SIRS) negative and SIRS positive colic horses at admission time (T0), then 24 (T1), 48 (T2), 72 (T3) and 96 (T4) hours after admission. Within row, different superscripts denote a significant difference (a ≠ b: $p < 0.05$).

**H**	**Total Colic Horses**										*SEM*
(n = 43)	*T0 (n = 48)*		*T1 (n = 38)*		*T2 (n = 35)*		*T3 (n=31)*		*T4 (n=23)*		
2.27^{a}	2.51^{b}		2.50^{b}		2.49^{b}		2.50^{b}		2.48^{b}		0.07
H	*SIRS-N*	*SIRS-P*	*SIRS-N*	*SIRS-P*	*SIRS-N*	*SIRS-P*	*SIRS-N*	*SIRS-P*	*SIRS-N*	*SIRS-P*	
(n = 43)	*T0 (n = 27)*	*T0 (n = 21)*	*T1 (n = 21)*	*T1 (n = 17)*	*T2 (n = 20)*	*T2 (n = 15)*	*T3 (n = 16)*	*T3 (n = 15)*	*T4 (n = 13)*	*T4 (n = 10)*	
2.27^{a}	2.48^{b}	2.49^{b}	2.47^{b}	2.46^{b}	2.47^{b}	2.54^{b}	2.52^{b}	2.51^{b}	2.55^{b}	2.55^{b}	0.066

H: healthy horses; SIRS-N: SIRS negative horses; SIRS-P: SIRS positive horses.
